# Annual Energy Usage Reduction and Cost Savings of a School: End-Use Energy Analysis

**DOI:** 10.1155/2014/310539

**Published:** 2014-11-18

**Authors:** Aiman Roslizar, M. A. Alghoul, B. Bakhtyar, Nilofar Asim, K. Sopian

**Affiliations:** ^1^Mechanical Engineering Faculty, Universiti Teknikal Malaysia Melaka, 76100 Melaka, Malaysia; ^2^Solar Energy Research Institute, Universiti Kebangsaan Malaysia, 43600 Bangi, Malaysia; ^3^School of Economy, Finance and Banking (SEFB), Universiti Utara Malaysia (UUM), 06010 Kedah, Malaysia

## Abstract

Buildings are among the largest consumers of energy. Part of the energy is wasted due to the habits of users and equipment conditions. A solution to this problem is efficient energy usage. To this end, an energy audit can be conducted to assess the energy efficiency. This study aims to analyze the energy usage of a primary school and identify the potential energy reductions and cost savings. A preliminary audit was conducted, and several energy conservation measures were proposed. The energy conservation measures, with reference to the MS1525:2007 standard, were modelled to identify the potential energy reduction and cost savings. It was found that the school's usage of electricity exceeded its need, incurring an excess expenditure of RM 2947.42. From the lighting system alone, it was found that there is a potential energy reduction of 5489.06 kWh, which gives a cost saving of RM 2282.52 via the improvement of lighting system design and its operating hours. Overall, it was found that there is a potential energy reduction and cost saving of 20.7% when the energy conservation measures are earnestly implemented. The previous energy intensity of the school was found to be 50.6 kWh/m^2^/year, but can theoretically be reduced to 40.19 kWh/mm^2^/year.

## 1. Introduction

With the steady economic growth in Malaysia since 2005, energy demand increased at an average rate of 6% annually from 2005 to 2010 [1]. The energy demand in buildings rose steadily in the past decade, despite the fact that the main energy resources such as oil and gas are running low [2]. It was predicted that the global energy consumption will rise by 1.6% annually between 2009 and 2030 [3]. The current state of global energy resource requires efficient energy usage so that the current resources will last until a suitable alternative is discovered [4]. Apart from the direct approach of finding alternative energy sources, an important aspect is managing the current energy supply in a wise and prudent manner. This is determined by looking at the energy efficiency of the buildings, which is determined through an energy audit. In this study, an energy audit will be conducted to determine the energy usage efficiency and potential energy reduction and cost savings for a primary school in Malaysia.

Generally, an energy audit is an exercise that is conducted to study the energy usage of a building or electrical equipment. It is a systematic method to determine how energy is being used in the building. From an energy audit, the total energy being used can be determined, and if required, the energy consumption of different equipment can be determined as well [5].

It is also possible to simulate the implementation of potential energy conservation measures that will improve the energy utilization in a particular building. An energy audit can also predict the possible energy savings and cost reductions from the efficient usage of energy [4]. Additional motivation towards the study of energy audits is emphasized after various governments around the world are starting to set standards of energy usage to increase the implementation of energy efficient buildings [6]. There are three types of energy audits that are usually implemented: preliminary audit, targeted energy audit, and detailed grade audit [7]. Generally, the technique that is to be used is determined by the complexity and the conditions of the intended energy analysis. For a preliminary audit, a mathematical model to predict the possible potential energy savings and cost reduction is deemed sufficient. A mathematical model to calculate the annual energy consumption was used to simulate some similar conditions to this research.

A few energy audit studies on schools have been conducted in many countries, which is indicative of interest in this topic around the globe. An energy audit was conducted by Alajmi [9] on an educational building in a hot climate country, the State of Kuwait. Although the heating, ventilation, and air conditioning (HVAC) system consumes the most energy within that building (87%), a similar energy audit technique was used where they gathered the information regarding its initial usage and compared it with the potential energy reduction that can be made. Several recommendations were made for energy conservation opportunities, which were categorized into nonretrofitting (no cost) and retrofitting (with cost). The research found that 6.5% of energy can be saved from the nonretrofitting recommendations, which are mainly changes in the schedule of the lighting and equipment usages. It was also shown that, by changing the type of lighting, it will enable them to save a further 2.3% of their energy usage. Ultimately, 52% of energy can be saved if all of the recommendations are implemented, including energy conservation opportunities that require additional costs.

Another study by Dall'O and Sarto was conducted on 49 school complexes consisting of 77 buildings for various usages [16]. The research was done in two phases, where the first phase aimed to gather data on the energy consumptions. In the second phase, energy conservation measures were proposed for three different scenarios: a standard scenario, a cost-effective scenario, and a high performance scenario. It takes into consideration the issue of the investment required to implement these energy retrofits and also their respective payback periods. It was found that energy savings of 15%, 67%, and 81% are possible from the implementation of these energy conservation measures, respectively. The research concludes that to implement a certain energy retrofit, a variety of factors needs to be considered, as they might be very difficult or not cost-effective in many cases. These factors can vary from the cost of retrofitting comparable to building new buildings, to changes in the number of population. It is also important to highlight that the study by Dall'O and Sarto focuses on the HVAC system of the buildings.

A research was also conducted on 62 buildings under the Serbia Energy Efficient Project, Phase 2, of which 28 were school buildings [17]. Their study focuses only on the heat consumption of the buildings, which is in line with the objective of improving energy efficiency. Although this differs greatly from our research, it is interesting to look at the results of conducting an energy audit and implementing energy efficiency improvements on the buildings. The research showed that they managed to realize an energy consumption reduction of 49% (average energy consumption of 128 kWh/m^2^/year) from the initial consumption of 252 kWh/m^2^/year.

There were also other studies that used similar methods of finding the potential energy usage reduction from the implementation of energy conservation measures compared to the initial energy consumption, despite the fact that almost all of the studies were either done for schools that were in different climates, or focused on different energy systems such as heating, ventilation, and air-conditioning, which differs from our condition. A study involving energy audit was conducted for 24 schools in Slovenia [10]. This study looks at the heat losses by the school in areas of air and water heating. With high heat losses, more energy will be consumed to replace the heat. The study shows that the heat losses of the school buildings are 89% higher than the recommended values. Another study on high schools in central Italy showed that there are potential energy savings of 46% when implementing some energy conservation measures [11].

Perez and Capeluto [12] also conducted a study for school buildings in hot-humid climates. The study uses simulation techniques to identify the potential energy savings and focuses on the thermal energy conservation measures. A high performance design for school buildings shows that there is a potential energy consumption reduction of more than 50%. However, this reported study differs from this study, as the climatic conditions are wildly different. The energy consumption by the schools in Malaysia focuses more on the electrical equipment instead of heating, ventilation, and air conditioning system. At the time of this study, there has yet to be any published work regarding schools with similar conditions to our study.

The aim of this study is to analyze the potential energy usage reductions and cost savings of schools in Malaysia. It covers a preliminary audit, which will indicate whether a more detailed audit is required. In this research, cases with the implementation of energy conservation measures will also be modelled. The author feels that this study is necessary and important to initiate benchmarking for energy consumption in schools, since education is an integral part of the development of a nation. Furthermore, the number of schools in Malaysia, accounting for both primary and secondary, totals up to about 10,101 schools [18]. Thus, the expense can be focused more on the education process instead of its infrastructure. This benchmark, which was previously unavailable, would help the stakeholders of the schools, especially the Ministry of Education, to build better energy efficient schools in the future and instill energy awareness among the pupils.

## 2. Analysis of Current Energy Usage

This research is conducted in two stages. The first stage involves the analysis of the current energy usage of the school, which is indicative of its efficiency, and whether improvements can be made. Meanwhile, the second stage involves the proposal of several energy conservation measures. This section explains the data collection methods and the mathematical equations used in this study.

### 2.1. Actual Energy Usage

Tables 1 and 2 show the background information and the electrical equipment that are used in the school. This information will be used to analyze the current usage of energy of the school by calculating the annual energy usage and its intensity.

Table 3 shows the energy usage costs that are obtained from the utility bills for 2011. This actual energy usage will then be compared with the calculated energy requirement of the school. It must also be pointed out that, in the middle of 2011, there was a change in the electricity tariff. From January to April, the electricity tariff was RM 0.397 per kWh. Starting from May, the electricity tariff increased to RM 0.43 per kWh. This electricity tariffs change was noted in the calculations. For this stage of the research, these two terminologies will be used.

- Actual energy usage: the electricity usage that was consumed by the school in 2011 based on the utility bills.
- Calculated energy requirement: the electricity usage that should be used by the school under efficient conditions, calculated from the collected information.

A worst case scenario, where the load factor is set to 1, will be used for calculating the calculated electricity requirement.

### 2.2. Calculating Energy Requirement

Comparing the actual energy consumption with the calculated energy requirement will show whether the energy usage for 2011 was indeed efficient. The efficient energy consumption by the electrical equipment in the school is calculated using the following equation, as used by Saidur et al. [4]:

(1) $$\begin{array}{c} {\text{AEC}}^{a}={\text{UH}}^{a}\times C^{a}\times {\text{LF}}^{a}, \end{array}$$

where AEC^*a*^ is the annual energy consumption of equipment *a* in kWh; UH^*a*^ the usage hours of the equipment *a*; *C* ^*a*^ the capacity or power rating of equipment *a*, in kW; and LF the loading factor of equipment *a*. The loading factor for all the electrical equipment was set to 1 to model a worst-case scenario.

The energy intensity explains the efficiency of the school in terms of energy usage per area. The energy intensity was calculated using the following equation, similar to Saidur et al. [4]:

(2) $$\begin{array}{c} \text{EI}=\sum \frac{\text{AEC}}{\text{TFA}}, \end{array}$$

where ∑AEC is the total annual energy consumption of the equipment in the school in kWh; TFA is the total floor area in square meters.

### 2.3. Results of First Stage Analysis

#### 2.3.1. Usage Hours of Electrical Equipment

The usage hours of the electrical equipment depend on the operating hours of the school. Generally, a school operates from Monday to Friday for teaching and on Saturday for extracurricular activities. In some cases, these extracurricular activities require the students to stay overnight at the school. The school operates 203 days for that school year, with varying operating days. For calculation of the electrical usage, an assumption was made that the usage hours of the electrical equipment follow the operating hours of the school. This will provide us with a worst-case scenario, which, when compared to the actual electricity usage, will show whether the school used its electricity in an efficient manner.  Table 4 shows the number of daily operating hours for different areas of the school.

#### 2.3.2. Energy Consumption by Electrical Equipment

**Figure 1:**
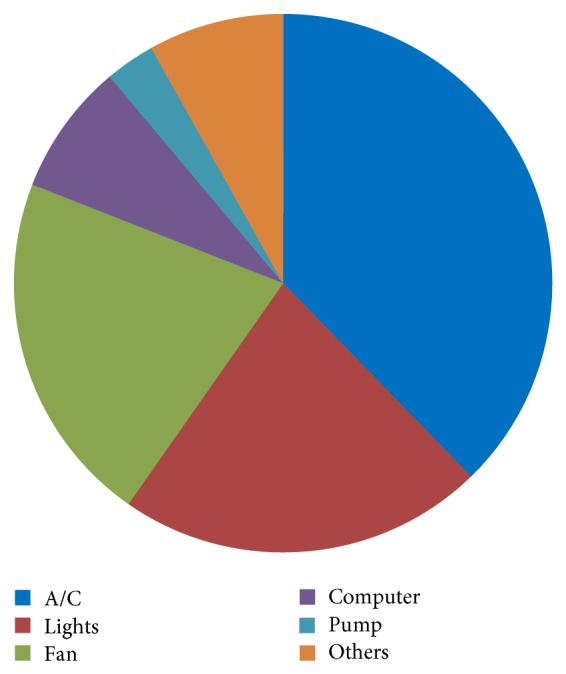
Distribution of electricity consumed by equipment in the school.

The distribution of electricity consumed by the equipment in the school is illustrated in Figure 1.  Table 5 shows the details of electricity usage by the top five energy consuming equipment types in the school. It can be observed that the air conditioning system consumes 38% of electricity. The lighting system follows at 22% and fans, at 21%. In any case, the energy conservation measures should focus on this high-electricity consuming equipment in order to give us a better chance at determining potential energy savings.

#### 2.3.3. Comparing Results

From the information being gathered, the actual energy usage and the calculated energy requirement can be compared, as shown in Table 6. It can be observed that the school used 60520.28 kWh, which exceeds its electricity requirements. The required electricity, which was calculated using (1), was found to be 53533.05 kWh. In this study, an assumption was made such that all the electrical equipment is operated at maximum loading factor. The electricity usage was also assumed to follow the listed operating hours. These assumptions overestimated the required electricity usage, but it was still less than the actual amount being used. The school incurred a minimum loss of 11.7% by using more electricity than necessary, at 6998.24 kWh. This amounts to a total bill of RM 2947.42 for that schooling year.

By improving the energy habits and practices of the users, the school would be able to reduce their energy usage and costs. Some energy usage practices that can be looked into are the habits of users, the operating hours, and the settings for the electrical equipment.

Considering the total energy usage and the total floor area, the energy intensity of the school was also compared in both actual and calculated cases. It can be observed that the actual energy intensity of the school is 50.6 kWh/m^2^, but it could have been 44.8 kWh/m^2^/year, with efficient energy usage practices.

## 3. Energy Conservation Measures

Energy reduction and cost savings can be further achieved by using energy conservation measures. The second stage of the study focuses on the lighting in the school, as it consumes 22% of the total energy. The energy conservation measures being proposed were based on the requirements of the MS1525:2007 standard for efficient energy nonresidential buildings in Malaysia [8].

### 3.1. Lighting System

The areas that consumed the most energy through the lighting system are the classrooms and the corridors.  Table 7 shows the illuminance standard for both classrooms and corridors.

The illuminance is affected by the quantity of lamps and their capacity. Using the Lumen method, the required number of lamps and their capacity can be determined from the following equation [19]:

(3) $$\begin{array}{c} E=\left(\frac{F}{A}\right)\times \text{CU}\times \text{LLF}, \end{array}$$

where *E* is the illuminance level required at the work surface, in lux; *F* the total number of lamps times the rated lumens of each lamp, lm; *A* the total area of the plane where the work is done; CU the coefficient of utilization, per unit; and LLF the light loss factor as a percentage of the rated lamp lumens, per unit. By knowing the illuminance level from (3), we can design an optimal lighting system. From calculations, we found the CU to be 0.68, while for cases where the maintenance plan is unknown, the LLF is set to 0.80 [20]. The factors that contribute to the overall design of the system are the type of light bulb, the quantity, and their arrangements.

Nevertheless, this equation is only accurate for the corridors, as the lights are only used at night, and thus does not take into consideration the daylighting effect. Energy conservation measures for the classroom are therefore proposed to focus on the operating hours instead of the setup of the lighting system. This is to consider measures that do not require any retrofitting cost. The experimental measurements of the illuminance in the classrooms were carried out to determine the best operating hours of the lights. This was done by taking measurements using a lux meter at several points in the concerned area.

Summarizing the lighting system, two energy conservation measures were proposed.

1. Corridors: redesign the setup of the lighting system—quantity of bulbs, location of the bulbs, and the type of bulbs.
2. Classrooms: improve the operating conditions of the lights in the classrooms—in terms of operating hours.

### 3.2. Air Conditioning System

The first stage of the study shows that 38% of the energy is consumed by the air conditioning system, as shown in Figure 1. This is almost double of the next highest energy consuming equipment. Previous studies proved that conducting energy study on HVAC system would be beneficial. Selecting a suitable system for building and operating it correctly has the potential of saving up to 20% of energy. In the case of Lam et al. [21], they shifted the chiller's load to the night via thermal storage, and up to 25% in the case of Fasiuddin and Budaiwi [22], where they simulated different HVAC systems while maintaining thermal comfort. Improving the operating settings and control strategies of the HVAC system also showed energy saving potentials. Mathews et al. [23] simulated several control strategies (air-bypass, reset control, setback control, improved start-stop times, economizer, and CO_2_ control) and found that a potential of energy savings of up to 66% can be achieved. A study by Kwong et al. [24] on air conditioned tropical buildings in Malaysia showed that raising the set-point temperature by just 2°C will result in total savings of 2150 GWh annually, which is equivalent to RM730 million.

This paper will only focus on the lighting system. Nevertheless, it should be pointed out that further energy studies should be carried out specifically on the air conditioning system to further increase energy savings in schools.

## 4. Energy Saved from Energy Conservation Measures

### 4.1. Lighting System

#### 4.1.1. Corridors

The current fluorescent lamps used in the corridors have a capacity of 36 W each. From (3), we found that the capacity of the lamps can be reduced to 15 W if the quantity is reduced from 50 pieces to 43 pieces to meet the requirement of 20 lux from the MS1525:2007 standard. The original and proposed lighting system configuration is shown in Table 8.

The new configuration gives a potential energy reduction and cost saving that is presented in Table 9. It can be observed from Table 9 that there is a potential energy reduction and cost saving of 64.6% from the original system. This is an energy reduction of 4459.2 kWh, which will save the school RM1855.20 compared to the original lighting system in the corridors. This is a significant saving, as it is more than half the initial energy consumption.

It can be said that the lighting system of the corridors is significantly overdesigned. By changing to a lower rated light bulb and rearranging the lights, an energy saving of 64.6% can be achieved.

#### 4.1.2. Classrooms

Measurements of the illuminance for six different classes located at the extremities of the school were taken using a lux meter at different time periods that reflects a normal school day. This is to understand the lighting conditions that are experienced in the class throughout its usage. The results of the measurements are presented in Table 10.

It can be observed that most classes would have reached the minimum requirement of 300 lux before 10.00 am. It is safe to assume that, on normal days, the lights in the classes can be switched off beginning from 10.00 am, as it already meet the minimum illuminance requirements from natural daylights.

**Figure 2:**
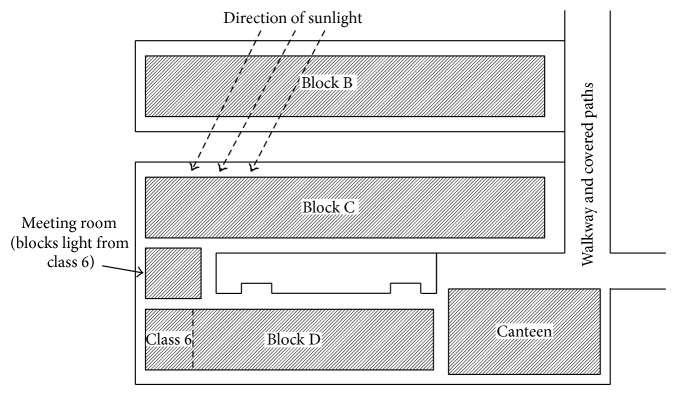
Partial view of the school's architectural layout.

Table 10 shows that class 6 hardly meets the requirement throughout the day. This is due to the design of the buildings, as the interconnected buildings block the sunlight from going into the class. The building blocking the lights is circled in Figure 2. Therefore, the results of the reading for this class were not used to propose new operating hours. Another study should be done to solve this particular lighting problem.

For the lighting inside the classes, it is proposed that the school changes the operating hours of the lights but maintains the type and design of light bulbs. The newly proposed operating hours are shown in Table 11. It can be observed that the operating hours were reduced to three hours a day, which will increase energy savings.

The potential energy reduction and cost saving for the new operating hours for the lighting systems in the classes were calculated and shown in Table 12. It can be observed that there is a significant reduction of 52.1% in the energy usage of the classes after the lighting system operating hours were changed. The energy usage of the lights in the classes can be reduced by 1030.86 kWh from the initial energy usage of 1978.02 kWh. This results in a cost saving of RM 427.32.

### 4.2. Combined Energy Usage and Cost Savings

Table 13 shows the summary of the potential energy reduction and cost savings from the improvements in energy usage practices and lighting system. It was found that a potential energy reduction and cost saving of 20.7% can be achieved from better energy practices and the lighting systems alone. This can be translated into an energy reduction of 12487.3 kWh/year and a cost saving of RM 5230.94/year. The energy reduction will contribute to a reduced energy intensity of 40.19 kWh/m^2^/year.

### 4.3. Comparing with Similar Studies

Table 14 shows the results of various energy studies carried out for schools for different conditions around the world. Although each piece of research has its own focus, it can be observed that our results are comparable to researches of similar nature. The energy conservation measure of improving energy usage practices and lighting system operations for our case will result in a potential energy usage reduction of 20.7%. Further studies on improving the efficiency of the air conditioning system in our school might also give comparable results to researches that focused on HVAC systems.

## 5. Conclusion

The actual energy used by the school was 60 520.28 kWh, which costs the school a total of RM 25 152.20, resulting in an energy intensity of 50.6 kWh/m^2^/year. It was determined that energy is being wasted via the operation of the electrical equipment in the school. This study showed that 11.6% energy reduction and cost saving can be achieved by implementing efficient energy usage practices. Apart from that, 9.1% energy reduction can also be achieved through the correct design and configuration settings for the lighting systems. Overall, this results in potential energy reduction and cost savings of up to 20.7%. The implementation of the mentioned energy conservation measures would also reduce the energy intensity from 50.8 kWh/m^2^/year to 40.19 kWh/m^2^/year, which is a significant achievement. Further energy studies on more schools in the country will provide us with a better understanding of energy usage conditions of schools in this country and open up more opportunities for potential energy savings.

## Figures and Tables

**Table 1:** Background information of the school.

Name of school	Sekolah Rendah Sri Al-Amin
Total floor area	1195 m^2^
Number of classrooms	23

**Table 2:** List of electrical equipment in the school.

Equipment type	Quantity	Power rating/equipment Watts (W)

A/C 1	14	1190
A/C 2	1	1250
A/C 3	1	1850
Amplifier	1	250
Desktop computer	23	200
Fan	137	75
Fax	1	100
Fridge	3	100
Kettle	2	1850
Lights	191	36
Microwave	1	800
Photocopier	2	1300
Printer	5	120
Pump 1	1	370
Pump 2	1	1500
Rice cooker	1	400
Stand Fan	1	50
TV	2	150
Wall Fan	4	35

**Table 3:** Energy usage from electricity bills.

Month	Electricity usage (kWh)	Cost of electricity usage (RM)

Jan.	4685.87	1860.29
Feb.	4870.45	1933.57
March	5116.51	2031.26
April	6202.20	2462.27
May	5732.89	2282.50
June	5044.07	2168.95
July	6584.00	2831.12
August	4596.00	1976.28
Sept.	5939.00	2553.77
Oct.	5205.00	2238.15
Nov.	4141.35	1780.78
Dec.	2402.93	1033.26

Total	60520.28	25152.2

**Table 4:** Number of operating hours for different areas in the school.

Type of area	Monday–Thursday	Friday	Saturday	Overnight
	usage hours	usage hours	usage hours	usage hours
Main office	8	8		
Primary school office	8	8	4.5	
Teachers room	8	8	4.5	
Treatment room	1 hour every school day			
Class	6.5	3.5	4.5	11
Directors room	3 hours every school day			
Library	1 hour every school day			
Nursery	7.5	4.5		
Hall	1	1		
Science laboratory	4			
Computer room	1 hour every school day			
Seminar room	once a week-2 hours			
Toilet	9	9	4.5	

**Table 5:** Top five energy consuming equipment types.

Rank	Equipment type	Electricity usage	Contribution to total energy consumption
		kWh	

1	A/C	20193.94	38%
2	Lights	11768.77	22%
3	Fan	11370.09	21%
4	Computer	4242.20	8%
5	Pump	1593.24	5%
6	Others	4353.8	6%

**Table 6:** Actual and calculated electricity usage and cost.

	Actual	Calculated

Electricity usage (kWh)	60520.28	53522.04
Cost (RM)	25152.20	22203.78

**Table 7:** Illuminance requirement from MS1525:2007 [8].

Area type	Illuminance

Corridor	20 lux
Classroom	300–500 lux

**Table 8:** Lighting system configuration that meets requirements.

Equipment	Original	Calculated

Fluorescent lamps	50 pieces of 36 W lamps	43 pieces of 15 W lamps

**Table 9:** Potential energy reduction and cost savings from the corridors.

	Electricity usage	Cost
	kWh	RM

Actual usage	6904.22	2872.94
After ECM	2446.02	1017.74

Reduction	4458.20	1855.20
Reduction Percentage	64.6	64.6

**Table 10:** Average illuminance (lux) in the class rooms at different times of the day.

Time of day	Class room					
	1	2	3	4	5	6

9 to 10	386	270	607	404	326	86
11 to 12	380	353	688	362	403	131
1 to 2	684	400	1239	651	645	217
3 to 4	270	297	720	283	263	45

**Table 11:** Suggested operating hours of the lighting system of the classes.

Time period	Operating hours	Total hours

Morning	8–10 am	2 hours

Afternoon	3-4 pm	1 hour

**Table 12:** Potential energy reduction and cost savings from the classes.

	Energy usage	Cost
	kWh	RM

Actual usage	1978.02	819.88
After ECM	947.16	392.56

Reduction	1030.86	427.32
Reduction Percentage	52.1	52.1

**Table 13:** Potential energy reduction and cost savings from energy conservation measures.

				Percentage Reduction	
		Energy usage	Cost	Energy usage	Cost
		kWh	RM	%	%

Actual usage		60520.28	25152.20	—	—

Potential reduction	(1) Energy usage practices	6998.24	2948.42	11.7	11.7
	(2) Lighting system potential reduction	5489.06	2282.52	9.1	9.1
	**Total**	**12487.3**	**5230.94**	**20.8**	**20.8**

**Table 14:** Energy usage reduction results for various researches carried out on schools.

Researcher	Energy usage reduction	Focus of energy conservation measures

Alajmi, 2012 [9]	8.8%	Nonretrofitting and lighting
Butala and Novak, 1999 [10]	89%	Heating
Desideri and Proietti, 2002 [11]	46%	Thermal and electrical
Perez and Capeluto, 2009 [12]	50%	HVAC and lighting
Santamouris et al., 2007 [13]	20%	Heating
Dimoudi and Kostarela, 2009 [14]	13.34%	Heating and cooling
Becker et al., 2007 [15]	(1) 28–30% for northern classroom orientations (2) 17-18% for southern classroom orientations	Passive building designs and ventilation schemes
Dall'O and Sarto, 2013 [16]	(1) Standard scenario, 15% (2) Cost-effective scenario, 67% (3) High performance scenario, 81%	HVAC
Bećirović and Vasić 2013 [17]	49%	HVAC, motors, passive building design

## References

[1] Chua S. C., Oh T. H. (2011). Green progress and prospect in Malaysia. *Renewable and Sustainable Energy Reviews*.

[2] Saidur R., Hasanuzzaman M., Yogeswaran S., Mohammed H. A., Hossain M. S. (2010). An end-use energy analysis in a Malaysian public hospital. *Energy*.

[3] Chua S. C., Oh T. H. (2010). Review on Malaysia's national energy developments: key policies, agencies, programmes and international involvements. *Renewable and Sustainable Energy Reviews*.

[4] Saidur R., Rahim N. A., Masjuki H. H., Mekhilef S., Ping H. W., Jamaluddin M. F. (2009). End-use energy analysis in the Malaysian industrial sector. *Energy*.

[5] Yik F. W. H., Yee K. F., Sat P. S. K., Chan C. W. H. (1998). A detailed energy audit for a commercial office building in Hong Kong. *HKIE Transactions Hong Kong Institution of Engineers*.

[6] Lee W. L., Yik F. W. H. (2004). Regulatory and voluntary approaches for enhancing building energy efficiency. *Progress in Energy and Combustion Science*.

[7] Saidur R. (2010). A review on electrical motors energy use and energy savings. *Renewable and Sustainable Energy Reviews*.

[8] Department of Standards Malaysia (2007). *Code of Practice on Energy Efficiency and Use of Renewable Energy for Non-Residential Buildings*.

[9] Alajmi A. (2012). Energy audit of an educational building in a hot summer climate. *Energy and Buildings*.

[10] Butala V., Novak P. (1999). Energy consumption and potential energy savings in old school buildings. *Energy and Buildings*.

[11] Desideri U., Proietti S. (2002). Analysis of energy consumption in the high schools of a province in central Italy. *Energy and Buildings*.

[12] Perez Y. V., Capeluto I. G. (2009). Climatic considerations in school building design in the hot-humid climate for reducing energy consumption. *Applied Energy*.

[13] Santamouris M., Mihalakakou G., Patargias P., Gaitani N., Sfakianaki K., Papaglastra M., Pavlou C., Doukas P., Primikiri E., Geros V., Assimakopoulos M. N., Mitoula R., Zerefos S. (2007). Using intelligent clustering techniques to classify the energy performance of school buildings. *Energy and Buildings*.

[14] Dimoudi A., Kostarela P. (2009). Energy monitoring and conservation potential in school buildings in the C′ climatic zone of Greece. *Renewable Energy*.

[15] Becker R., Goldberger I., Paciuk M. (2007). Improving energy performance of school buildings while ensuring indoor air quality ventilation. *Building and Environment*.

[16] Dall'O G., Sarto L. (2013). Potential and limits to improve energy efficiency in space heating in existing school buildings in Northern Italy. *Energy and Buildings*.

[17] Bećirović S. P., Vasić M. (2013). Methodology and results of Serbian energy-efficiency refurbishment project. *Energy and Buildings*.

[18] Malaysia Ministry of Education (2013). *Statistics: Number of Schools*.

[19] Rea M. S. (1993). *Lighting Handbook*.

[20] Mitroy J. (2007). Methods for calculating illumination. *Acoustic and Lighting*.

[21] Lam J. C., Li D. H. W., Cheung S. O. (2003). An analysis of electricity end-use in air-conditioned office buildings in Hong Kong. *Building and Environment*.

[22] Fasiuddin M., Budaiwi I. (2011). HVAC system strategies for energy conservation in commercial buildings in Saudi Arabia. *Energy and Buildings*.

[23] Mathews E. H., Botha C. P., Arndt D. C., Malan A. (2001). HVAC control strategies to enhance comfort and minimise energy usage. *Energy and Buildings*.

[24] Kwong Q. J., Adam N. M., Sahari B. B. (2014). Thermal comfort assessment and potential for energy efficiency enhancement in modern tropical buildings: a review. *Energy and Buildings*.

